# “Learning Science Is About Facts and Language Learning Is About Being Discursive”—An Empirical Investigation of Students' Disciplinary Beliefs in the Context of Argumentation

**DOI:** 10.3389/fpsyg.2017.00946

**Published:** 2017-06-08

**Authors:** Patricia Heitmann, Martin Hecht, Ronny Scherer, Julia Schwanewedel

**Affiliations:** ^1^Institute for Educational Quality Improvement (IQB), Humboldt-Universität zu BerlinBerlin, Germany; ^2^Department of Psychology, Humboldt-Universität zu BerlinBerlin, Germany; ^3^Centre for Educational Measurement (CEMO), Faculty of Educational Sciences, University of OsloOslo, Norway; ^4^Leibniz Institute for Science and Mathematics Education at Kiel University (IPN)Kiel, Germany

**Keywords:** argumentation, beliefs, disciplinary school culture, language education, science education

## Abstract

Argumentation is considered crucial in numerous disciplines in schools and universities because it constitutes an important proficiency in peoples' daily and professional lives. However, it is unclear whether argumentation is understood and practiced in comparable ways across disciplines. This study consequently examined empirically how students perceive argumentation in science and (first) language lessons. Specifically, we investigated students' beliefs about the relevance of *discourse* and the role of *facts*. Data from 3,258 high school students from 85 German secondary schools were analyzed with multigroup multilevel structural equation modeling in order to disentangle whether or not differences in argumentation across disciplines exist and the extent to which variation in students' beliefs can be explained by gender and school track. Results showed that students perceived the role of *facts* as highly relevant for science lessons, whereas discursive characteristics were considered significantly less important. In turn, *discourse* played a central role in language lessons, which was believed to require less knowledge of *facts*. These differences were independent of students' gender. In contrast, school track predicted the differences in beliefs significantly. Our findings lend evidence on the existence of disciplinary school cultures in argumentation that may be the result of differences in teachers' school-track-specific classroom practice and education. Implications in terms of a teacher's role in establishing norms for scientific argumentation as well as the impact of students' beliefs on their learning outcomes are discussed.

## Introduction

Science education is aimed at fostering a variety of students' competencies, which concern not only the mere acquisition of knowledge but also skills that help students communicate and evaluate scientific knowledge (Baram-Tsabari and Osborne, 2015). Some of these skills are discipline-specific (e.g., planning a scientific experiment in science lessons), whereas others are rather discipline-general (e.g., reading). In order to describe and understand discipline-specific learning processes, argumentation is considered important because it is needed to formulate reasoned justifications or ideas and, thus, to cope with the increasing complexity of knowledge within a discipline (e.g., Driver et al., 2000; Berland and Hammer, 2012). Engaging in argumentation means constructing and supporting claims by using evidence and reasoning abilities as well as questioning, challenging, and revising one's own and others' claims, evidence, and reasoning (Osborne, 2010; Berland and Hammer, 2012). Evidently, argumentation is an important transversal key competence across all disciplines.

However, the way in which argumentation is taught in lessons might feature discipline-specific characteristics as shown by a recent study on students' written argumentation skills in science and language (Heitmann et al., 2014). One reason for these varying characteristics of argumentation may lie in so-called disciplinary school cultures, which can be described as patterns of thinking, perceiving, and doing. Disciplinary school cultures shape students' and teachers' beliefs about what is a “correct,” “authentic,” or “accepted” argument in a concrete discipline. According to (Borg, 2001, p. 186), a belief is a “[…] proposition which may be consciously or unconsciously held, is evaluative in that it is accepted as true by the individual, and is therefore imbued with emotive commitment; further, it serves as a guide to thought and behavior.” Thus, such beliefs play a central role in learning processes and reflect the manner in which a discipline is typically conceptualized and presented in school settings (Hericks and Körber, 2007). Students may hold different beliefs about the understanding and practice of argumentation in the disciplines, and independent of their cognitive abilities, these beliefs may have a serious impact on students' ability to argue. Differences in beliefs across disciplines can be interpreted as evidence of existing disciplinary school cultures in argumentation.

Given that argumentation is a broad construct, we focused on two important characteristics: *facts* and *discourse*. *Facts* are central to the process of argumentation because they serve as a basis for supporting an argument with data and evidence. *Discourse* relates to communication processes, provides the means for debating or argumentative discussions, and represents the discursive aspect of argumentation. Concerning students' beliefs about *facts* and *discourse*, it makes sense to ask about the extent to which these beliefs are determined by individual and contextual factors. Some studies have suggested that girls and boys hold different beliefs about science and language because language learning, for instance, is often characterized as “soft” or feminine (Willems, 2007), whereas science is considered to be “hard” or masculine (Hannover and Kessels, 2002). In addition, the school tracks that students attend might influence their beliefs about *facts* and *discourse*. This may be due to the differences in teachers' beliefs about argumentation and the educational practices they apply in the classroom, both of which can be the result of different teacher study programs (de Brabander, 2000). As a consequence, variation in students' beliefs about *facts* and *discourse* may be explained by gender and school track.

Against this backdrop, the goal of the current research is to investigate the extent to which students' perceive specific aspects of argumentation and whether these beliefs are subject to differences across the two school disciplines of science and language^1^. Our primary goal is to examine whether there are differences in students' beliefs about argumentation in these two domains and to determine the extent to which gender and school track shape these differences. Current curricula in nearly all western countries request promoting argumentation in different disciplines. To ensure a systematic competence development in argumentation that enables students to recognize and apply argumentation across and within disciplines, the clarification of students' beliefs about argumentation in different disciplines is helpful. Beliefs influence classroom practices. Thus, knowledge about disciplinary beliefs can be a starting point for the design and the improvement of learning and teaching argumentation in the classroom, for explaining disparate argumentative behavior in the disciplines, and for providing information on potential learning barriers.

## Theoretical framework

### Disciplinary cultures

The existence and relevance of differences between (scientific) disciplines such as chemistry, sociology, or linguistics has been discussed in various fields of research (e.g., Becher, 1987; Huber, 1990). Empirical research generally supports the view that there are important differences between disciplines or disciplinary groupings (Becher, 1987; Neumann et al., 2002; Multrus, 2005). Each discipline clearly has its own particular characteristics and qualities, and of course these are not purely epistemological. Disciplines are also cultural phenomena: They are embodied in collections of like-minded people, each with their own codes of conduct, sets of values, and distinctive intellectual tasks (Becher, 1981). In this context, the term *disciplinary culture* is used to describe a common set of assumptions, attitudes, conceptualizations, epistemologies, and values held by members of a discipline (e.g., Becher, 1987; Huber, 1990). Disciplinary cultures cover the tradition of a discipline, particularly its fields of knowledge and research, methodologies, practices, scientific issues, and how the results are represented and interpreted (Multrus, 2005). The culture involves the thought patterns, evaluation, and behavior of the individuals who belong to a specific discipline (Green and Dixon, 2002). The traditions of the discipline shape its members and this is again expressed in a professional perception and view of the world (e.g., Kelly and Chen, 1999; Multrus, 2005). Thus, the members of a discipline (e.g., scientists in a particular field, members of a classroom) affiliate over time and create particular ways of talking, thinking, acting, and interacting (Green and Dixon, 2002).

### Characteristics of disciplinary school cultures

The concept of disciplinary cultures can also be transferred to the school setting. In this setting, the concept comprises teachers' and students' beliefs about a discipline and how these beliefs influence classroom practices. Disciplinary school cultures reflect the manner in which the disciplines are typically conceptualized and treated in school settings. In the following, unless otherwise noted, the term “disciplines” refers to school settings and covers school subjects such as science, mathematics, history, and language.

In general, beliefs about learning or the nature of a discipline are cultivated through education in school (Wang et al., 2016). The school is often the first contact students get to a discipline. Students and teachers develop ingrained beliefs about the characteristics of a discipline, and these beliefs influence their behavior in terms of, for example, learning and teaching objectives, common and useful knowledge, ways of learning and teaching, students' performances, difficulty of content, popularity, and problems (Bastian and Combe, 2007; Hericks and Körber, 2007). These beliefs results in discipline-specific differences in mindsets (Trautmann, 2005). According to Müller-Roselius (2007), it is important that a common belief exists, regarding, an unquestioned acceptance of the expectation of what counts as “correct,” “authentic,” or “accepted” ways of learning and teaching in reference to disciplinary school cultures. Thus, disciplinary school cultures can be defined as social constructs that refer to the “rules” that are cultivated in school lessons and that describe students' and teachers' commonalities in thinking or questioning, among others (Hericks and Körber, 2007; Müller-Roselius, 2007). These beliefs are taken for granted and make implications about what counts as scientifically “good,” sophisticated, efficient, or typical (Yackel and Cobb, 1996; Carlone, 2004; Partanen and Kaasila, 2015).

Concerning students' beliefs about the facets of knowledge, knowing, and beliefs students are exposed to in their learning environments, researchers often refer to an “epistemic climate” (e.g., Muis and Duffy, 2013). Teachers' beliefs, in particular, play an important role in a school's epistemic climate. For example, in the discipline of science, Kind (2016) stated that “science teachers' (…) beliefs about science may influence those of their students” (p. 9). Jones and Leagon (2014) moved this statement even further into the context of argumentation by claiming that teachers' beliefs about science argumentation are key for promoting the classroom practice of science argumentation. These classroom practices may in turn influence students' beliefs about science argumentation and science in general (Bell and Linn, 2002). Contributing to this line of reasoning, McNeill and Knight (2013) uncovered the relation between teachers' beliefs in science argumentation and how they were educated in K-12 instruction with respect to promoting science argumentation. Whereas, elementary teachers were more likely to connect argumentation in science to similar practices in other disciplines such as language and mathematics, high school science teachers were more likely to focus on the scientific content of argumentation (McNeill and Knight, 2013).

However, it is important to note that disciplinary school cultures cannot be considered to be “pure” disciplinary cultures in the sense of academic disciplinary cultures. In a school setting, a subject typically involves less complexity in its contents and methods than at universities. In contrast to disciplines at universities, school contexts are, for example, not designed to produce new scientific knowledge (McDonald and Kelly, 2012). In addition, teachers pursue educational goals in order to best help students to understand science, and such goals are not identical to the practices that help scientists develop new scientific understandings (McDonald and Kelly, 2012). Consequently, science classroom learning practices might not be completely authentic in this way.

### The concept of argumentation

Despite disciplinary differences, existing conceptualizations of argumentation share a common idea, that is, argumentation represents a process through which people engage in proposing, criticizing, and evaluating ideas that are debatable (e.g., Sampson and Clark, 2008). Hence, argumentation is important in order to reveal *why* people think their position is “reasonable” (Kuhn, 1993). For an argument to appear reasonable, grounds have to be stated that show which position an arguer takes toward a debatable idea and how this position is elaborated (Kuhn, 1993; Winkler, 2003). This demand comes along with engaging in argumentative processes and is based on the assumption that claims are deniable (Kuhn, 1993; Walton, 1996).

At the same time, however, various frameworks of argumentation exist that add discipline-specific perspectives on aspects of the argumentative process. In science education, for instance, Toulmin's Argumentation Pattern (Toulmin, 1958) is a prominently applied framework. This framework focuses on the structure of an argument which is characterized by the presence of specific structural components such as claim, data, warrant, backing, and rebuttal. At the same time, several alternative frameworks of argumentation developed to evaluate the content and quality of informal arguments exist (e.g., Walton's dialogue theory; for a review of argumentation frameworks, please refer to Nussbaum, 2011). For example, the content-oriented perspectives on the quality of an argument focus on the underlying content and thus the application of reliable knowledge that has been gained from texts, tables, diagrams, results of experiments, or other sources to undermine an opinion (for a systematic review on assessing arguments in science education, see Sampson and Clark, 2008). In language education, further frameworks focus on linguistic features of an argument as indicators of, for instance, students' knowledge about how to formulate justifications or counterarguments (Winkler, 2003; Feilke, 2010). In light of these different foci on argumentation, it is of interest whether students share common beliefs about argumentation across different school lessons, and, consequently, which disciplinary school culture of argumentation students form.

### Characteristics of a disciplinary school culture of science argumentation

Science and technology influence our society in various ways and shape a significant part of our cultural identity. Science education in schools is therefore aimed at fostering scientific literacy, which enables students to participate in society by developing the ability to communicate about technical developments and scientific research (e.g., Bybee, 1997; NGSS Lead States, 2013). In particular, students should gain insights into scientific phenomena, which serve as a means of discovering the world and understanding human nature. They should also be able to understand and apply scientific language, comprehend its history, and communicate scientific results. Likewise, students should learn about scientific methods in order to have the ability to acquire knowledge and discuss its limitations.

On this point, there are various kinds of characteristics that are central to learning science. The current paper focuses on two characteristics that are highly relevant to learning science and, at the same time, are immanent within scientific argumentation processes: (a) the acquisition of *facts* or the role of *facts* and (b) the engagement in *discourse* on debatable aspects of knowledge (i.e., discursive characteristics). It is important to note that both characteristics can be considered to be two central facets of argumentation and cannot capture the intricacy of argumentation as a higher-order thinking skill.

In acquiring *facts*, students should use knowledge about basic principles in science and apply them toward arguing about or explaining scientific questions and understanding or evaluating scientific and socioscientific issues. In doing so, science lessons are often characterized by a hypothesis-based approach toward understanding or simulating the scientific process of obtaining data, theories, or concepts (Kuhn et al., 2008). In our study, this characteristic of science lessons is referred to as *facts*. Of course, this represents only one type of knowledge relevant for science lessons among others such as procedural or epistemic knowledge. In terms of scientific literacy, students should learn how *facts* in science evolve over time and thus learn about the (un)certainty of knowledge (e.g., Bybee, 1997; NGSS Lead States, 2013). *Facts* are also immanent in the process of argumentation in science because *facts* support an argument's claim and are components of data and evidence amongst other elements (e.g., Osborne, 2010). Thus, *facts* are important for providing justification. In fact, without argument, the construction of reliable (and also revisable) knowledge would be impossible (Osborne, 2010).

The aspect of *discourse* represents another important but not contrary characteristic of scientific literacy. Science learning is also aimed at engaging students in discourse and interpretive processes (Kelly and Chen, 1999). *Discourse* is a mechanism of communication that is central for justifying, questioning, or evaluating *facts*, theories, and concepts in science (Osborne, 2010). Scientists debate the merits of alternative scientific theories and models, and review one another's work as an integral part of the scientific disciplinary culture. Moreover, discursive abilities are important for formulating ideas, debating justifications, and talking about science with others in science lessons (Olitsky, 2007). In the classroom, students develop a sense of what constitutes a sophisticated or acceptable scientific contribution in order to avoid unproductive discussions (Partanen and Kaasila, 2015).

The process of argumentation focuses on claims that are supported by evidence (on the basis of *facts*) and require the ability to compare, contrast, and distinguish different lines of reasoning with warrants, backings, and so forth (e.g., Osborne, 2010). It is of particular interest to link pieces of evidence with claims and other elements in order to formulate a scientifically adequate argument. It is evident that, next to the role of *facts, discourse* is another important characteristic of a scientific argument. *Facts* can be seen as a relevant component of a scientific argument, whereas *discourse* constitutes a mechanism of communication within the argumentation process.

However, science is often presented in schools in a “ready-made” form (Latour, 1987), which may lead to the perception of *facts*, and thus, static or undebatable knowledge. In this context, students believe their teachers expect them to act in completely goal- and fact-oriented ways because their work is based on observations and the conclusions drawn from them (Decke-Cornill and Gebhard, 2007). As a consequence, science lessons are often associated with a well-defined body of knowledge and skills to be taught and are perceived as narrowly defined with a strong scientific orientation (e.g., adherence to the rules of good scientific practice, correct use of scientific terms; Grossman and Stodolsky, 1995; Decke-Cornill and Gebhard, 2007). Along these lines, science is constructed as a precise, clearly defined discipline that deals with “pure” ideas (Becher, 1981), and, due to an accumulation of *facts*, it is also regarded as a discipline of “objective truth” (Hericks and Körber, 2007; Willems, 2007). Such beliefs may result in an overemphasis of *facts* in science lessons. This in turn offers only limited opportunities for students to actively contribute to science lessons and therefore to express their scientific personality or identity (Hannover and Kessels, 2002). Science lessons may at the same time be characterized by a lack of discourse at the expense of what we know (Osborne, 2010).

### Characteristics of a disciplinary school culture of language argumentation

Mastering language is an essential educational goal in order to understand, find order in, and shape the world. The national educational standards for language education in Germany, inter alia, have formulated the aims of enabling students to handle social demands on the basis of values and norms, to cope verbally with situations, to share their thinking with others (e.g., argue, express feelings, or ideas), and to develop the ability to handle criticism (KMK, 2004). Altogether, language education is aimed at contributing to the development of students' personality (i.e., sense of identity), the ability to share one's sentiments, which involves positioning oneself as an autonomous person, and an awareness of how to handle oneself (Willems, 2007). In the long run, this contribution should increase students' self-confidence, social skills, and ability to work in teams (KMK, 2004). Against this background, it is noteworthy that especially qualitative analytic approaches and hermeneutic-interpretative statements tend to dominate language lessons (Decke-Cornill and Gebhard, 2007).

Language lessons are also characterized by the (a) role of *facts* and (b) the application of *discourse*. The acquisition of *facts* is relevant because students need information about language (e.g., grammar, discourse markers), literature (e.g., different kinds of genres, structure of texts), media (e.g., different representations, characteristics of information, and entertainment functions), and the topic at hand. For instance, students should know how to structure an argument and also support their arguments with relevant information about a debatable topic; that is, they need to address relevant *facts* (e.g., Winkler, 2003). Besides verifiable evidence, values, or norms, arguments can be built on individual examples or personal experience as long as students present this “subjective evidence” reasonably (Krelle, 2014).

Because methods in language lessons often require an individual approach that leaves room for interpretation, students also need discursive abilities in order to take a position, reflect on the material, and negotiate a consensus (KMK, 2004; Willems, 2007). To achieve this, students should develop skills such as the aptitude to contribute to discussions, the capacity for empathy, or the ability to avoid making a hasty judgment (Willems, 2007). Concerning argumentation, it is pivotal to follow a line of reasoning that consists of, among others, realizing the debatable character of a problem, gathering information, developing and weighing alternative options, and coming to a conclusion or consensus while also considering values and norms (e.g., KMK, 2004; Willems, 2007). In this context, great emphasis is placed on a precise linguistic integration of arguments (Feilke, 2013).

According to Willems (2007), language lessons tend to strongly emphasize personal arguments and can affect students' willingness to adapt their answers to new perspectives. This may result in the downgrading of the importance of specific *facts*. The content can thus be utilized to achieve these aims, which may be due to the fact that the content and the medium of communication are often congruent (Willems, 2007). This strong focus on communicational and linguistic aspects often leads to the belief that language education is a “soft,” subjective, and emotional discipline (Decke-Cornill and Gebhard, 2007; Willems, 2007).

To conclude, argumentative skills related to *facts* and *discourse* are important characteristics for learning in both disciplines (i.e., science and language) but are often treated differently in school lessons in these different disciplines, thus potentially leading to distinct disciplinary school cultures.

### Gender differences in students' discipline-specific beliefs

There is some evidence that students' belief about a discipline, which influences their attitudes or behaviors in the classroom in terms of learning or interest, differs across gender. Hannover and Kessels (2002) suggested that students' expectations and attitudes toward (the characteristics of) a discipline not only depend on their performance, self-concept, and motivation but are also shaped by the image of a particular discipline. This image is based on socially shared knowledge and reflects stereotypes or prototypes that are associated with the discipline (Hannover and Kessels, 2002). Their study showed that secondary school students perceived science as having strong connotations of gender; in particular, about half of the students considered physics to be a subject for boys (Hannover and Kessels, 2002). Moreover, a study conducted by Willems (2007) revealed that teachers often characterized boys as more determined and result-oriented than girls. In particular, the successful acquisition of “hard” knowledge (i.e., *facts*) with supposedly objective truth in physics requires characteristics that are often attributed to or preferred by boys (Willems, 2007; Archer et al., 2010). Accordingly, boys should be attached to the role of *facts*.

By contrast, teachers tend to depreciate boys' discursive abilities and instead attribute characteristics such as being a good listener and loving to discuss and exchange views to girls (Willems, 2007). Teachers even tend to assume that girls are naturally gifted with discursive abilities that are independent of their socialization through language education (Willems, 2007). Given that the discipline of language is strongly associated with these skills, it is often characterized as “soft” or feminine (Willems, 2007). Consequently, girls should be attached to *discourse*. However, there are also opposing views on gender differences in the context of discourse and the ways students choose to engage in discourse (e.g., in argumentation). Because argumentation often incorporates competition, which implies disagreement and possibly consternation, it can be hypothesized that female students will consider this competition less attractive than boys and will thus lose interest in science (McDonald and Kelly, 2012). In this line of thinking, girls should be less attached to *discourse*. Nevertheless, because some studies have indicated that teachers make different attributions to their students with respect to the two aspects of disciplinary school culture, this may also affect students' beliefs about the relevance of these characteristics.

### Potential sources of teachers' disciplinary beliefs

Besides explaining differences in students' disciplinary beliefs at the individual level, contextual factors in school may also play an important role in shaping these beliefs. In fact, Archer et al. (2010) argued that the development of students' scientific identities and beliefs about science strongly depends on the context in which learning takes place.

One potential contextual source of these beliefs involves teachers' education and professional development. Haerle and Bendixen (2008) pointed out that teachers' epistemological beliefs are related to their professional development and vary across different teacher training programs. Similar conclusions were drawn in several other studies (e.g., Sadler et al., 2006; Zohar, 2008). Besides the effects of professional development, the university and college cultures in which preservice teachers are educated shape their beliefs about knowledge and the nature of disciplines (Muis and Sinatra, 2008). For instance, this was confirmed in a study by Trautwein and Lüdtke (2007) who found differences in university students' general and discipline-specific epistemological beliefs across different academic environments. Specifically, the authors found that students who were enrolled in mathematics, science, and engineering believed that scientific knowledge was certain to a larger extent than students who were enrolled in the social sciences. It can therefore be hypothesized that teacher education conveys beliefs about knowledge and the nature of disciplines to preservice teachers, and this in turn might then account for the ways teachers constitute disciplinary school cultures (Grossman and Stodolsky, 1995).

In fact, the German programs for educating future academic and nonacademic track teachers differ greatly^2^, particularly with respect to the number of courses in general pedagogy and their length, subject-specific education, and the subject domains that preservice teachers are offered. Specifically, academic track teachers spend considerably more time studying the actual subject domains such as science or language, whereas pedagogy and educational sciences are in the main focus of nonacademic track teacher education—which is a very typical situation in Germany (e.g., Müller et al., 2008; Kleickmann and Anders, 2013). These differences are a result of the different mandates that academic and nonacademic track education has. Whereas, academic track education is oriented toward developing scientific understanding and competence in order to prepare students for university, nonacademic track education is aimed at providing students with a general, rather practical education that prepares them for the working world (e.g., Baumert et al., 2010; Blömeke, 2016). Given these differences across school tracks, differences in beliefs about science and science argumentation between the teachers enrolled in these two programs may occur.

There is some evidence that teachers with the highest level of teacher education perceive science as an extremely “hard” and highly specialized discipline, whereas language learning represents a “softer” and less specialized discipline to them (de Brabander, 2000). The qualifier “hard” refers to the role of *facts* that is characterized as testable, objective, and established in contrast to everyday knowledge. Hence, our assumption is that students in academic tracks should be more attached to the acquisition of *facts*, especially in the science subjects (Willems, 2007).

Bringing together the main arguments presented in this section—teachers' beliefs are shaped in teacher education at university, may differ with respect to their type of education (e.g., academic vs. nonacademic track), and may affect students' beliefs—it is reasonable to expect students' beliefs about argumentation in science and language learning to vary across school tracks. The current study consequently sought to examine these differences.

## Research questions

The goal of the present study was to examine students' beliefs about two central characteristics of argumentation and investigate whether these beliefs differ across the two disciplines of science and language. Students' beliefs within both disciplines were of interest with a focus on (a) the role of *facts* and (b) the relevance of discursive abilities (i.e., *discourse*). On the one hand, these two aspects can be regarded as two relevant characteristics of learning and on the other hand as two elements that are relevant in the argumentation process in both disciplines. If students' beliefs vary across the two disciplines, this can be interpreted as evidence for the existence of disciplinary school cultures in science and language. The knowledge about existing disciplinary school cultures in turn is relevant for shaping disciplinary and interdisciplinary learning processes in schools, and for developing a reasonable understanding of argumentation in different academic disciplines.

Against this backdrop, we examined three research questions concerning the existence of disciplinary school cultures and their relation to students' and schools' characteristics. The first research question addresses the extent to which students perceive differences between the two disciplines:

(1) Are there differences in students' beliefs concerning the roles of *facts* and *discourse* between science and language lessons?

In order to describe these differences, we assessed students' beliefs with rating scales and gathered qualitative information from open-ended questions on the relevance of *facts* and *discourse* for argumentation in science and language lessons. Expanding on these differences, Research Questions 2 and 3 investigated school-level variation and the influence of gender and school track on students' beliefs. As described earlier, beliefs about argumentation or specific subjects are shaped by students' learning environment and might therefore be subject to variation therein (Archer et al., 2010). Given that schools vary in the extent to which curricula—both in the domain of science and first-language learning—are enacted or in the extent to which teachers are trained in order to shape adequate beliefs about these domains (e.g., Haerle and Bendixen, 2008), Research Question 2 is primarily concerned with quantifying this variation for students' beliefs about the relevance of *facts* and *discourse*. Research Question 3 takes a step further by focusing on two potential sources that might explain student- and school-level variation in these beliefs.

(2) To what extent are differences in beliefs about the relevance of *facts* and *discourse* related to the schools the students attend?
(3) How can the differences in students' beliefs be explained on (a) the student level and (b) the school level?
  - To what extent differ students' beliefs about the relevance of *facts* and *discourse* in science and language lessons related to their gender?
  - To what extent differ students' beliefs about the relevance of *facts* and *discourse* in science and language lessons related to the school track they attend?

## Method

### Participants

This study focused on students at the end of the lower secondary level in Germany. Participants were 3,258 students in 85 schools. A two-stage sampling procedure was applied. In the first stage, eight federal states were chosen in a way that they form a representative sample of all 16 German federal states. For instance, the sample consisted of urban city states as well as states with large rural areas from several geographical regions. In the second stage, schools within each state and two 10th-grade classrooms within these schools were randomly selected. The resultant sample used in this study was thus as representative for the population of grade 10 German students as possible. Students' average age was 15.5 years (*SD* = 0.7 years) and ranged from 14 to 18 years. About half of the students were female (51.3%).

Because the German school system differentiates between tracks, students attended different school types, of which 45.2% were enrolled in an academic school track (*Gymnasium*), that is, the highest educational track across all federal states, and the other students attended nonacademic school tracks (e.g., *Realschule*).

### Data collection

Data collection took place as part of the German national educational assessment program at the end of the lower secondary level. It was administered in eight federal states and conducted by the Institute for Educational Quality Improvement (IQB). For schools, participation was mandatory; yet, students' participation was not. Although students were encouraged to participate (participation rate was 79.6%), they had the opportunity to withdraw their participation. The study has been approved by the ethics committees of the Federal Ministry of Education in Germany and the IQB. Consent was given by all relevant parties and participants. Data collection was anonymous. The assessment program included a questionnaire on sociodemographic information. The investigation of students' beliefs was integrated into this part of the assessment. Students worked on items that referred to the relevance of *facts* and *discourse* in science and language lessons (quantitative measure). In addition, students had to answer an open-ended question about the relevance of these aspects for argumentation in science and language lessons (qualitative measure).

### Study measures

#### Quantitative measure of disciplinary school cultures

In order to assess students' beliefs, we administered a questionnaire that consisted of six items (see Supplementary Material S1). Students had to rate these items on a 5-point Likert-type scale ranging from 1 (*strongly disagree*) to 5 (*strongly agree*). The items addressed two scales: The first scale comprised three items that referred to the perceived role of *facts* in science and language lessons; it was therefore labeled *facts*. The following sample item illustrates the content of this scale: “In science/language lessons, it is important to use technical terms for descriptions or justifications.” The second scale comprised three items and was labeled *discourse*. It covered students' beliefs about the importance of formulating one's own standpoint toward controversial issues in school lessons, for example, “In science/language lessons, I am expected to express my opinion about controversial issues.” Parallel item wordings were used in order to minimize methodological bias, which can occur with different item formulations across disciplines. The specific items can be found in the Supplementary Material S1.

Table S1 in the Supplementary Material shows the reliability indices for both scales in both disciplines. We computed McDonald's ω (McDonald, 1999) from a confirmatory factor analysis with freely estimated factor loadings in the statistical software M*plus* 7.11 (Muthén and Muthén, 1995–2013). McDonald's ω represents a measure of internal consistency that outperforms the commonly used coefficient of Cronbach's α (Trizano-Hermosilla and Alvarado, 2016). The reliability was satisfactory for all subscales (i.e., *facts* and *discourse*) across subjects (i.e., science and language), as McDonald's ω ranged from 0.71 to 0.85. Support for the distinction between *facts* and *discourse* within the two disciplines was derived from the item correlations (see Supplementary Material Tables S3, S4). For both the science and language scales, items had homogenous correlations within a factor (black-rimmed box) that were much higher than the item correlations between factors. For instance, item correlations for science *facts* ranged from *r* = 0.52 to 0.60, whereas item correlations across factors ranged from *r* = –0.04 to 0.02.

#### Qualitative measure of disciplinary school cultures in argumentation

In addition to using the quantitative measure, we collected students' beliefs about the roles of *facts* and *discourse* for argumentation in science and language lessons in an open-ended item format. The instructions read as follows:

Sometimes you have to formulate a point of view on a controversial issue; that is, you need to generate an argument about a topic in the science subjects (biology, chemistry, and physics) and in language lessons.
Explain what you think is expected of you if you make an argument in science lessons in comparison with making an argument in language lessons.
You can provide examples to illustrate your opinion.

In the present paper, the qualitative data from the open question were used to supplement the quantitative results from the rating-scale questionnaire. We analyzed a subsample of the open answers qualitatively to identify central beliefs about the relevance of *facts* and *discourse* in argumentation in the two disciplines. The development of an exhaustive category system and the systematic analysis of the open questions is reported elsewhere (Schwanewedel and Heitmann, in preparation).

### Statistical analyses

In order to address our research questions, we tested a total of six models in M*plus* using the robust maximum likelihood (MLR) estimator and continuously treated items. This estimator ensures correct standard errors in cases in which deviations from the normality of observations occur (Kline, 2015) and Likert scales with at least four response categories are used (Beauducel and Herzberg, 2006). Rates of missing data were less than 2% for each item, and the full-information-maximum likelihood procedure was used to handle them.

The purpose of the first four models was to investigate the measurement invariance of the two constructs *facts* and *discourse* between the two disciplines science and language at the individual (student) level, treating the disciplines as “grouping variables.” This analysis was particularly important for addressing our research questions because we needed to ensure that the measurement of the two constructs was comparable across disciplines. If, in fact, sufficient levels of invariance are not given, differences in students' responses between subjects might be due only to differences in the ways in which the items are perceived (e.g., Rutkowski and Svetina, 2014). This measurement bias may compromise the comparability of the quantitative measures across disciplines. We specified two-group confirmatory factor analysis models in which the two disciplines represented the groups, across which different parameter constraints were imposed. In the first model, the structure of the measurement model was the same in the two disciplines, but the model parameters (e.g., factor loadings, item intercepts) were not constrained across disciplines (*configural invariance*). To identify this model, the factor loading of the first manifest item was set to 1 for each construct, and the means of the latent variables in both disciplines were set to the mean of the means of the manifest items (i.e., 4.39 for *facts* and 2.99 for *discourse* in science; 3.65 for *facts*, and 3.96 for *discourse* in language). In the second model, the factor loadings were constrained to be equal across disciplines (*weak invariance*). In the third model, item factor loadings and intercepts were constrained to be equal across disciplines (*strong invariance*). This implies that the mean of one of our two constructs could be freely estimated; we (arbitrarily) chose to freely estimate the means in language, whereas the means for the science scales remained fixed. Equal factor loadings and equal item intercepts across disciplines formed the prerequisite for comparisons of the factor means across the two disciplines (Research Question 1). In the fourth model, the error variances of the manifest variables were additionally constrained to be equal across disciplines (*strict invariance*).

Building on the strong measurement invariance model (Model 3), we additionally introduced schools as another level in order to address Research Question 2. Given that students reported on their perceptions of argumentation in science and language lessons in schools, a multilevel structural equation modeling approach was taken to approach the research questions. In fact, Marsh et al. (2012) and Wagner et al. (2016) suggested that, in contexts where students' perceptions of school- or classroom-based constructs are assessed, both the individual (i.e., student) and the aggregated level (i.e., schools or classrooms) need to be taken into account. The resulting model (Model 5) represented a two-group (science and language), two-level (students and schools) confirmatory factor analysis model with two latent factors (*facts* and *discourse*). This model allowed us to calculate the intraclass correlation coefficient (ICC), which indicates the portion of the variation that is due to the clustering of students in schools. Factor loadings were additionally constrained to be equal across levels. School weights were used to account for unequal numbers of students in schools. This model formed the basis for answering our first and second research questions. In a final model (Model 6), we added students' gender as a predictor at the student level (0 = *boy*; 1 = *girl*) and school track as a school-level predictor (0 = *nonacademic track*; 1 = *academic track*). This model formed the basis for addressing Research Questions 3a and 3b. Following(Enders and Tofighi, 2007) recommendations, we centered students' gender on the schools' means (i.e., group-mean centering) and school track on the overall sample mean (i.e., grand-mean centering). Broadly speaking, this kind of centering facilitates the interpretation of the effects on the different levels.

## Results

### Measurement invariance testing and model fit

Table S2 in the Supplementary Material presents the RMSEA and CFI-values for the six models. We chose these two fit indices because they are less sensitive to model complexity and sample size than other indices (Cheung and Rensvold, 2002; Chen, 2007). For the measurement invariance models, the differences in the CFI and RMSEA values from the previous model were computed. As a rule of thumb, the more parsimonious model should be chosen if ΔCFI exceeds.01 (Cheung and Rensvold, 2002; Chen, 2007). The least restrictive configural invariance model (Model 1) fit the data very well (CFI = 0.99, RMSEA = 0.035). The weak invariance model (Model 2) fit equally well (ΔCFI = 0.00). Model fit decreased just slightly when the intercepts were additionally constrained to be equal (strong invariance, Model 3, ΔCFI = 0.01). A substantial drop in model fit was observed for Model 4 in which the error variances were additionally constrained to be equal. Thus, strict measurement invariance was not given, and Models 5 and 6 were based on Model 3. The lack of strict measurement invariance was not problematic because strong measurement invariance was sufficient for our analyses. Overall, all models, except for Model 4, fit the data well.

We have divided the presentation of the results on our research questions into two subsections: First, we will address the first research question concerning the existence of disciplinary school cultures of argumentation in science and language lessons (Model 5). We will also include qualitative data from students' answers to the open-ended question to support or differentiate the results from the quantitative analysis. Second, we will report the results for Research Questions 2 (Model 5) and 3 (Model 6) to investigate the differences in *facts* and *discourse* in relation to schools and the covariates gender (student level) and school track (school level).

### Disciplinary school cultures in science and language lessons

#### Facts

**Figure 1:**
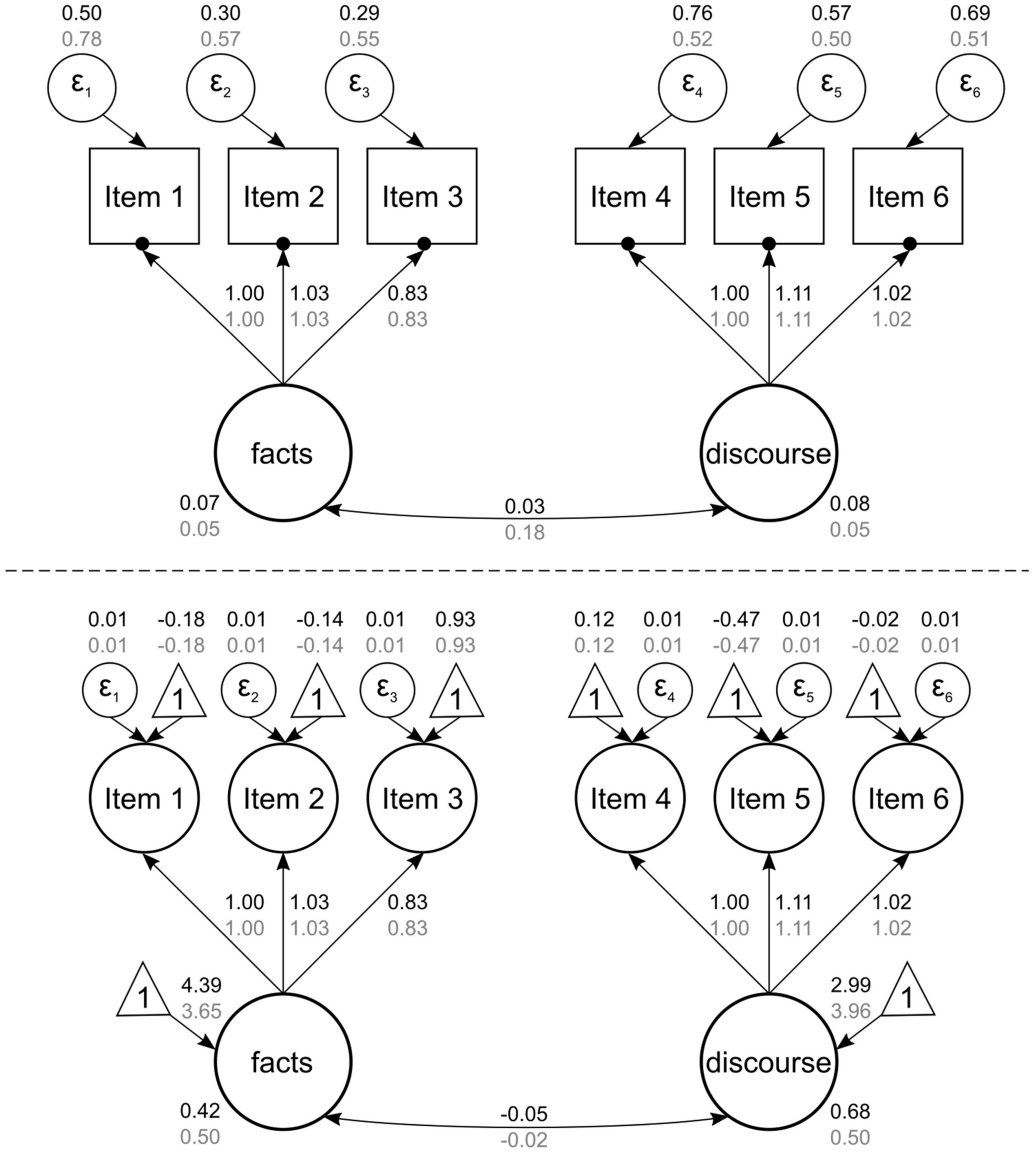
Multilevel model with factor means for *facts* and *discourse* (Model 5). Numbers in black refer to science; numbers in gray refer to language. The figure shows the unstandardized estimates. Factor means are the path coefficients ranging from the triangles to the latent variables. Double-headed arrows for variances have been omitted for the sake of clarity.

To address the first research question about whether there were differences in how science and language were perceived with respect to *facts*, we compared the differences in this construct between the two disciplines (see Figure 1, bottom; *student* level of Model 5). The mean for the construct *facts* in science was *M* = 4.39, whereas the mean in language was *M* = 3.65; this resulted in a difference of –0.74 (*p* < 0.001), which corresponds to an effect size of Cohen's *d* = –1.08 (calculated with pooled student variances). Science lessons were therefore perceived as much more strongly focused on *facts*. Students reported believing that they need knowledge or skills to memorize *facts* or technical terms in order to succeed in science lessons. Nevertheless, they also reported believing that *facts* are relevant for language lessons, too, but to a lesser extent. This difference in the relevance of *facts* in the two disciplines was also evident in students' written answers to the open questions, as the following examples show:

Student A^3^:

Scientific topics are grounded in approved facts; hence, they are not subjective. How good you can discuss is due to how much you know. In language lessons, everything is subjective; it is grounded in one's own experiences as a human being and how you view a topic. Consequently, there are many different solutions and not “only the correct one” though.

Student B:

In language lessons, you can enforce your arguments much better than arguments in science because there are specific rules and facts in science argumentation that cannot be changed by arguments.

Student C:

In my opinion, I have to argue with the help of technical terms and technical expressions in chemistry, physics, and biology. In language lessons, I can paraphrase words and I have far more options for expressing my personal opinion.

These responses show that students tended to consider the role of *facts* in scientific argumentation as static, approved, objective, and not in doubt. Of special importance were the correctness and the use of technical terms. These students believed that scientific argumentation heavily depends on the role (and the quality) of *facts*. Moreover, the nature of scientific evidence leads to a rather static way of engaging in argumentation because, in their opinion, these scientific *facts* seem to determine the process and/or the product of argumentation. In contrast to their answers for science, students highlighted the subjective character of *facts* in arguments in language lessons. In their opinion, evidence in a language argument is often based on one's own experiences or feelings, which in turn leads to more options for the process or product of argumentation. In addition, students felt that they had the option to paraphrase words, that is, they did not necessarily have to use specific technical terms.

#### Discourse

In order to analyze how science and language were perceived with respect to *discourse*, we compared the difference between the disciplines in a manner that was analogous to what we applied to assess *facts*. It is interesting that a different picture emerged (see Figure 1). Language lessons (*M* = 3.96) were considered much more focused on discussions about debatable issues than science lessons (*M* = 2.99) with a difference of 0.97 (*p* < 0.001) and *d* = 1.27. The students believed they needed many skills in order to make critical arguments and to ask questions in language lessons, which were perceived as far more focused on discussions. This trend is well-illustrated by the following three examples:

Student D:

If I argue in science lessons, I'll only have to justify why an issue/reaction/etc. is like it is and does not react in another way. An actual argument is not needed. One's own opinion does not matter. There is only one correct solution. In comparison, in language lessons, everybody can have their own opinion about a topic, which will then be discussed with one's classmates. Here, argumentation means you need to make your point of view clear by explaining issues and by trying to communicate your own opinion as persuasively as possible.

Student E:

In science lessons, it's all about finding the correct answer, for instance, for questions about cells, etc. In my opinion, in language, one can play out one's “freedom” better during argumentation. What you think counts. Of course, in the science subjects, too, but you have to argue scientifically.

Student F:

In the subject of language, everybody has their own right and wrong; everybody has their own thoughts and beliefs! Since everybody expresses their own view, there is no right and wrong! But in chemistry, for example, there is mostly one right answer, even if one has a completely different opinion/view.

These examples were quite prototypical of students' open answers on the relevance of *discourse* in the two disciplines. Students reported believing that discursive elements are a primary component of language lessons. Discussing debatable topics is connected with different valid options in this discipline, which students described as arguing “freely.” By contrast, a large number of students reported believing that there is only one correct answer in scientific arguments so that they perceived them as rather ready-made with no or few options to interact. Moreover, some students believed that forming an opinion is not part of science and, thus, there is no room for argumentation in the science subjects at all. Even though the mean for *discourse* for science was substantially lower than the mean for language, it was still about 3 (*M* = 2.99). This suggests that discursive elements are not entirely absent in science, as is also pointed out in the statement of Student E.

Furthermore, there were also students who compared the roles of *facts* and *discourse*, as the following example illustrates:

Student G:

I assume that one has to prove an assertion with, for example, equations or experiments in the science subjects. For language, I can instead justify my assertions with knowledge that I have or knowledge from books in different media. For me, a scientific argument is content-related and requires facts as well as an opinion, which is based on facts. I think in language, an argument is actually the forming of an opinion. It is more individual-related because in my opinion, everybody can have another viewpoint, whereas the sciences are based on facts.

The student compared the role of *facts* and *discourse* in the two disciplines but expressed a similar belief: Argumentation in science is based on *facts*, and, as a consequence, there is no choice to have different viewpoints. By contrast, argumentation in language was considered to be more open because knowledge is available from many resources such as students' pre-existing knowledge. Thus, *facts* seemed to be more a feature of science lessons and discursive characteristics of language lessons.

### Gender and school track differences in disciplinary school cultures

To answer Research Question 2 on the extent to which differences in *facts* and *discourse* were related to the schools, we disentangled schools and students (see Figure 1, top; *school* level of Model 5). We calculated the intraclass correlation (ICC) for each discipline (science, language) and construct (*facts, discourse*). The resulting ICCs were 0.15 for science on the *facts* factor, 0.09 for language on *facts*, 0.10 for science on *discourse*, and 0.09 for language on *discourse*. This implies that, for example, 15% of the variance in *facts* in science lessons was due to different schools; the beliefs depended to this extent on the school that students attended. Thus, there was substantial variation between schools.

**Figure 2:**
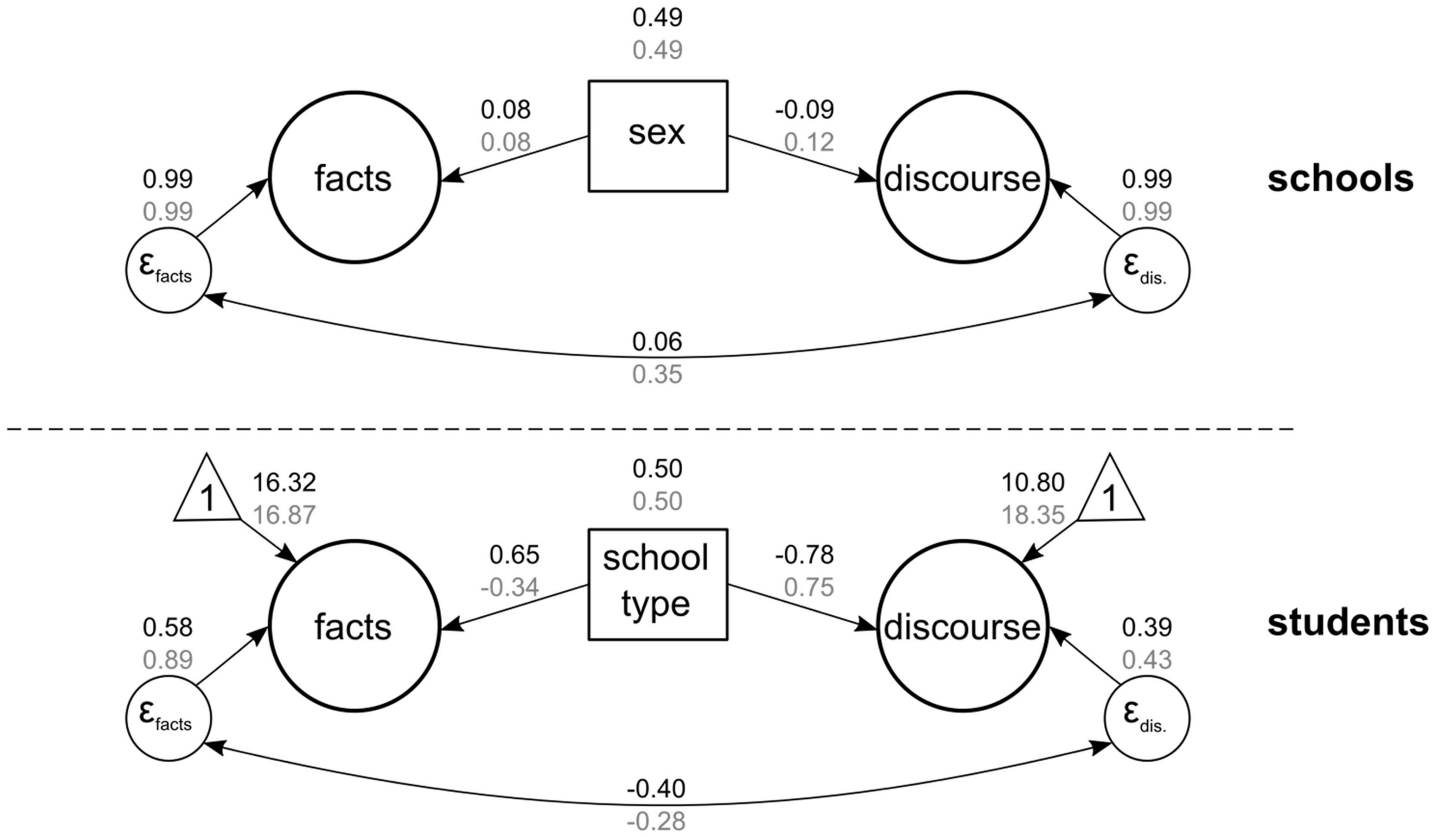
Multilevel model with gender and school track as predictors (Model 6). Numbers in black refer to science; numbers in gray refer to language. The figure shows the standardized estimates. Gender (0 = boys, 1 = girls; group-mean centered) and school track (0 = nonacademic, 1 = academic school track; grand-mean centered) were dichotomously coded. The measurement model corresponds to that shown in Figure 1.

To explain these differences between students and differences between schools (Research Questions 3a and 3b), we added students' gender and school track as predictors (see Figure 2) to explain variations in students and schools with the ICCs. The effects of gender were significant (all *p*s < 0.001) but rather marginal in size, as indicated by small amounts of explained variance: 0.08 for science on *facts* (*R*^2^ = 0.006, i.e., 0.6% of the variation in students in *facts* in science was explained by gender), –0.09 for science on *discourse* (*R*^2^ = 0.008), 0.08 for language on *facts* (*R*^2^ = 0.006), and 0.12 for language on *discourse* (*R*^2^ = 0.014). However, school track was a strong and significant predictor of school differences. For science on *facts*, the track effect was 0.65 (*p* < 0.001), indicating that students in academic school tracks were to a large extent oriented toward *facts* in science lessons. The corresponding explained variance was *R*^2^ = 0.424, that is, 42.4% of the variation in schools in *facts* in science was explained by school track. Furthermore, science was regarded as much less focused on *discourse* in academic school tracks (−0.78, *p* < 0.001, *R*^2^ = 0.614). For language, the opposite picture emerged: Language lessons in academic school tracks were perceived as much more oriented toward *discourse* (0.75, *p* < 0.001, *R*^2^ = 0.566) than in nonacademic school tracks. Moreover, *facts* were perceived as less relevant in language lessons in academic school tracks (–0.34, *p* < 0.001, *R*^2^ = 0.115).

The results suggest that students had a different understanding of the use and relevance of *facts* and *discourse* in the two disciplines. These differences in both aspects can be interpreted as characteristics of existing disciplinary school cultures and could be an explanation for students' varying argumentation practice, which will be discussed below. In addition, our analyses showed that the differences in students' beliefs were independent of gender. The analyses further showed that school track was a good predictor of differences in students' beliefs at the school level. Differences between the disciplines were especially apparent for students in an academic school track. Academic track students perceived science as more and language as less *fact*-focused than nonacademic track students, whereas for *discourse*, it was the other way around. That is, students in academic tracks tended to perceive science as less and language as more *discourse*-oriented compared with nonacademic track students.

## Discussion and implications

Our study was motivated by the question of whether students' beliefs about two central characteristics of argumentation—*facts* and *discourse*—would differ between science and language and what aspects would influence their beliefs. In the following, we first discuss the roles of *facts* and *discourse* with a special focus on science, and thus, what students consider adequate for scientific argumentation as well as how this may affect their argumentation practice. Second, we argue how gender and school track with a special view on German training programs and the role of teachers may contribute to disciplinary school cultures. Third, limitations and suggestions for future research as well as ongoing studies will be outlined.

### Disciplinary school cultures concerning the role of *facts*

Our findings revealed that students' beliefs about the role of *facts* in argumentation vary between the disciplines of science and language. In science, students more often reported believing they were expected to simply memorize *facts* and focus on using technical terms, whereas the role of *facts* in language was perceived as much less relevant. These results suggest that disciplinary school cultures concerning the role of *facts* in argumentation exist. Reasons for these existing disciplinary school cultures may be found in the nature of *facts*, which differs between the two disciplines. In order to help students acquire a basic understanding of complex scientific topics (e.g., the greenhouse effect or the genetic modification of plants), the presented *facts* are often limited or simplified to certain aspects and perspectives (i.e., didactical reduction) and a well-defined body of knowledge (Grossman and Stodolsky, 1995; Decke-Cornill and Gebhard, 2007). This in turn may create the impression in the science classroom that science content is rarely controversial and that there is only one correct answer to scientific questions (see Hericks and Körber, 2007; Willems, 2007). This corresponds to the fact that science is often presented in schools in a “ready-made” form (Latour, 1987).

Moreover, studies on epistemological beliefs have shown that students often have a rather limited perspective on science (e.g., Conley et al., 2004). Conley et al. (2004), for example, found that students often hold naïve conceptions of the characteristics and development of scientific knowledge and consider it to be certain and rather static. This picture of the nature of knowledge in science has parallels with students' beliefs about the essential role of *facts* in scientific argumentation in our study. Whereas, epistemological beliefs refer to beliefs about the nature of knowledge in general or concerning academic disciplines, disciplinary beliefs refer to concrete classroom situations and patterns of thinking, perceiving, and doing. Prospectively, the relations between epistemological beliefs and beliefs about concrete classroom situations in a discipline are worth investigating. As a desideratum, we need to ask whether epistemological beliefs are influenced or shaped by disciplinary school cultures or vice versa.

### Disciplinary school cultures concerning the role of *discourse*

Compared with *facts*, the opposite picture was revealed for *discourse*. Students considered *discourse* to be a crucial characteristic of argumentation in language and far less relevant in science argumentation. Because there are different beliefs in the two disciplines, it can be concluded that disciplinary school cultures of argumentation exist.

Kelly and Chen (1999) argued that students' appropriation of scientific discourse is related to how the teachers frame the activities and the social practices established in the classroom. Given that the classroom culture in science mostly includes repetitive tasks (e.g., verification processes in laboratory activities, defining terms) and is characterized by instruction that is based on the idea that teaching is the mode of transmission (Carlone, 2004), it makes sense that we found that students perceive science as rarely discursive. It might be reasonable to assume that teachers' instruction and classroom activities in science lessons influence students' beliefs. By contrast, students considered language lessons to be much more focused on discussions about debatable issues than science lessons. This result also appears to be consistent with the results of other studies as well as general aims promoted by language education (e.g., Winkler, 2003; KMK, 2004; Willems, 2007). Nevertheless, students did not consider *discourse* to be absent from science. Elements of discussion in science should thus be enhanced even more.

We then need to ask whether these rather stereotypical disciplinary beliefs are positive for teaching and learning argumentation. In a previous study, Heitmann et al. (2014) found that students produced less elaborated arguments in science and tended to write fragmented, one-sided argumentations in science, whereas language argumentations were far more elaborate and discursive. We would hence expect that perceiving science as not very discursive might negatively influence students' quality of argumentation. However, it would be worth thinking about whether the discussion of characteristics of arguments in language promotes the teaching and learning of argumentation in science.

### Gender and school track differences in students' disciplinary beliefs

To address possible gender effects in disciplinary school cultures, we investigated the extent to which students' gender influenced their beliefs about *facts* and *discourse*. Our findings revealed that the effect of students' gender was negligible; that is, girls and boys had the same beliefs of each characteristic in both science and language. Consequently, the assumption that girls should be attached to *discourse* (especially in language) and boys to *facts* (especially in science) was not confirmed by our study. Our data suggest that other predictors are more appropriate for describing disciplinary school cultures. Even if teachers may have gender-connoted beliefs as (Willems, 2007) study showed, these do not influence girls' beliefs about the relevance of the two characteristics of argumentation in school lessons compared with boys' beliefs. Of course, we cannot rule out the possibility that gender differences may occur for other characteristics of disciplinary school cultures. Further research will be necessary to explicitly investigate whether gender differences exist, and this may also have an effect on the gender-connoted image of the disciplines (see Hannover and Kessels, 2002).

A different picture emerged for the influence of the school track that students attended. Here, the expectation that students' beliefs would differ by type of school (i.e., academic vs. nonacademic) was confirmed. This finding may have a number of explanations and consequences: First, the disciplinary school cultures were more pronounced in academic tracks such that students perceived science as a “hard” discipline and language as “softer.” Because these beliefs are congruent with what has been found for teachers' beliefs in academic tracks (de Brabander, 2000), it seems possible that students' disciplinary beliefs may evolve at least in part from teachers' disciplinary beliefs. Currently, there is no substantial research on argumentative classroom practices in Germany, especially with regard to different school tracks. Future studies should therefore shed light on this line of research and investigate how many argumentative discussions occur during school lessons. Furthermore, it would be beneficial to know how *facts* and *discourse* become evident in argumentation and to survey its connection to teachers' beliefs with special regard to school tracking.

Second, teachers' academic track education is heavily oriented toward acquiring knowledge and competencies in science in an attempt to make them content specialists so that they can prepare students for university (Blömeke, 2016). This content-focused mandate of teachers' academic track education seems to influence students' beliefs about the relevance of *facts* and *discourse*, which are perceived as more relevant for science education compared with the beliefs of students from nonacademic tracks. Again, disciplinary beliefs appear to be related to students' teachers and thus the teachers' education. Consequently, the role of the way science is taught at universities and its consequences on teachers' beliefs on argumentation should be analyzed in more detail. It might be that science taught at universities can be interpreted as static and dogmatic and thus is distant from the formal goals of science education in schools. Moreover, students' stereotyped beliefs about science and language can have serious effects on young peoples' career choices. In future studies, it would be interesting to investigate if students' beliefs are linked to the relative low interest in science careers.

Third, another source of school track differences may lie in teachers' perceptions of students' level of ability, which is higher in academic track education. In a qualitative study of 40 teachers, Zohar et al. (2001) found that teachers' beliefs about low-achieving students were associated with their beliefs about the instruction of higher order thinking skills such as scientific argumentation. Specifically, almost half of these teachers believed that higher order thinking was not appropriate for teaching low-achievers. A good 10 years later, Sampson and Blanchard (2012) confirmed this finding and consequently argued that teachers' perceptions about students' ability levels create barriers to the integration of argumentation into science lessons. Given that a number of studies have revealed that students' average achievement levels in science, mathematics, and reading differ significantly across tracks (e.g., Pant et al., 2013), these ability differences may have an impact on teachers' perceptions of students, their instruction in general, and the ways in which they foster the development of scientific argumentation skills specifically. As a consequence, the degree to which teachers incorporate argumentation into their lessons could differ across school tracks and in turn influence students' beliefs about argumentation.

The complexity of teachers' beliefs in this area was further demonstrated in a more recent study in which practicing secondary school teachers completed a questionnaire that tapped into teachers' beliefs about high critical thinking activities and low critical thinking activities for high- and low-advantaged students (Warburton and Torff, 2005). The findings showed that teachers rated both high and low critical thinking activities as more effective for high-advantaged learners than for low-advantaged learners. Another study conducted by Katsh-Singer et al. (2016) supported these findings in a sample of 34 teachers. The authors further found differences in teachers' beliefs about the importance of *discourse* across high, middle, and low socioeconomic status students. Henceforth, we argue that these differences in students' beliefs may be interpreted in light of the role of the teacher, who represents the discipline in school and consequently plays a central role in establishing the norms of scientific and language argumentation in classrooms. As Yackel and Cobb (1996) argued, normative understandings (e.g., scientific arguments) are continually regenerated and modified by the students and the teacher through their ongoing interactions. So the influence of teachers in contributing to students' beliefs should be analyzed in detail, a topic that needs further attention in research on scientific argumentation. Future studies will need to shed light on this line of research and gather information about teachers' beliefs and instructional practices with a special focus on the relevance of *facts* and *discourse*. Furthermore, the socialization of teachers during their university studies may also be interesting to evaluate with a special look at teacher training in academic and nonacademic tracks.

### Implications and directions for future research

We introduced items to measure students' beliefs of the relevance of *facts* and *discourse* in science and language education. A lot of thought and experience went into the development of these items, so we strongly encourage other researchers to use them as well (see the Supplementary Material S1). Nevertheless, researchers should be aware of two limitations: First, in order to improve the reliability of the measures of *facts* and *discourse* in both disciplines, more items need to be developed. Second, although the psychometric properties were satisfactory in our sample, there is no guarantee for this high level of psychometric quality in other studies. One issue is that the items were administered in German, and thus, the English translations might not work as well as the original German items. Still, we are confident that we provided a sound first attempt that can be refined or extended in the future.

Moreover, it is unclear whether science is constructed as a homogenous discipline by students or whether they tend to differentiate between the disciplines of biology, chemistry, and physics, which are traditionally taught separately in German schools. A study by Multrus (2005) showed no differentiation between the three disciplines for disciplinary cultures at universities. All of them could be clustered into a complex of engineering-nature-medicine-economy (vs. an education-culture-social affairs cluster; Multrus, 2005). However, Multrus did not determine whether the disciplines were perceived as distinct in a school setting, so this question needs to be investigated further.

Another point to consider is that disciplinary school cultures and argumentation both appear to be broad multifaceted constructs, so items that capture facets other than *facts* and *discourse* should be developed. In an ongoing study, Schwanewedel and Heitmann (in preparation) are developing an exhaustive system for categorizing responses by applying a systematic analysis of the open question used to supplement the quantitative results from the rating-scale questionnaire. This includes various aspects of argumentation such as the adequacy of language, which includes, for example, the use of discourse markers and the layout of a text (e.g., length, fragments vs. continuous text). Different beliefs about the aim of an argument (persuasion vs. self-clarification; see Winkler, 2003) may be another facet of argumentation where disciplinary school cultures appear.

Finally, future studies should also investigate the impact of students' beliefs on their learning outcomes. The question of interest is: Do the disciplinary beliefs about argumentation promote or hinder students' learning outcomes? Therefore, students' arguments and the quality of their arguments (e.g., aspects of content, structure, and language) should be evaluated and linked with their beliefs about argumentation in the different disciplines. A detailed analysis of this relationship might provide valuable information about non-cognitive aspects of learning and explain the different argumentation strategies for science and language found in a previous study (see Heitmann et al., 2014).

The results of the current study may be fruitful for laying a conceptual and empirical foundation for research on teaching and learning with regard to argumentation and may be used to promote discussion about the relevance of students' beliefs in different disciplines. There is growing interest in analyzing whether discipline-specific beliefs influence the quality of an argument, especially for teachers who need information about how to teach their students to produce adequate arguments. Accordingly, a challenging area of future research would be to identify if and how students' beliefs can be affected in order to have an effect on their argumentation practices. That is, it may be reasonable to focus on the role of teachers and the development of appropriate interventions that can be applied to promote students' framing of their arguments. Regarding implications for the teaching and learning of argumentation in the classroom, approaches using metacognitive elements in the sense of discussing characteristics of an argument might be worth performing (e.g., Schworm and Renkl, 2007). A guiding question for such an approach could be “What makes a good argument in general, in science, and in language education?” The commonalities as well as discipline-specific characteristics of argumentation could be discussed among students, science teachers, and language teachers in order to make common representations of “good” scientific arguments and, thus, how to argue in order to be successful in science.

## Author contributions

Conception and Design: PH, MH, JS. Acquisition of Data: PH, MH. Analysis and Interpretation of Data: MH, RS. Drafting the Article: PH, MH. Revising it for Intellectual Content: PH, MH, RS, JS. Final Approval of the Completed Article: PH, MH, RS, JS.

### Conflict of interest statement

The authors declare that the research was conducted in the absence of any commercial or financial relationships that could be construed as a potential conflict of interest.

## Footnotes

^1^In our study, German is the students' first language, which will subsequently be referred to as the discipline of “language.”

^2^Schools in the German educational system are divided into school types that qualify students to attend technical colleges and universities versus vocational training programs. The former includes the German grammar school (*Gymnasium*) and some parts of comprehensive schools (*Gesamtschule*). The latter includes all other secondary schools (e.g., *Realschule*) but also primary school (*Grundschule*). German teacher training is differentiated in study programs for academic school types (*Gymnasium*) and nonacademic school types.

^3^Note that the students' responses were translated from German into English.
