# Supplementary material for: “Learning Science Is About Facts and Language Learning Is About Being Discursive”—An Empirical Investigation of Students' Disciplinary Beliefs in the Context of Argumentation
Source: Front Psychol. 2017 Jun 8;8:946. doi: 10.3389/fpsyg.2017.00946 (PMC5462966; doi:10.3389/fpsyg.2017.00946)
Supplement: Supplementary file 1 [file DataSheet1.docx]

**Supplemental Material**

Table S1. *Reliability Indices of the Facts and Discourse Scales*

|  | McDonald’s ω |
| --- | --- |
| *Facts,* science | .80 |
| *Facts,* language | .71 |
| *Discourse,* science | .85 |
| *Discourse,* language | .83 |

Table S2. *Model Fit for the Six Models*

| Model | Parameters | CFI | ΔCFI | RMSEA | ΔRMSEA |
| --- | --- | --- | --- | --- | --- |
| 1 Configural invariance | 38 | .99 | - | .035 | - |
| 2 Weak invariance | 34 | .99 | .00 | .032 | -.003 |
| 3 Strong invariance | 30 | .98 | .01 | .049 | .017 |
| 4 Strict invariance | 24 | .92 | .06 | .090 | .041 |
| 5 Multilevel | 38 | .97 | - | .038 | - |
| 6 Multilevel with predictors | 46 | .97 | - | .033 | - |

*Note.* ΔCFI and ΔRMSEA refer to the differences in the CFI and RMSEA values of adjacent models.

Table S3. *Interitem Correlations for Science*

|  | Item 1 | Item 2 | Item 3 | Item 4 | Item 5 | Item 6 |
| --- | --- | --- | --- | --- | --- | --- |
| Item 1 |  |  |  |  |  |  |
| Item 2 | .55 |  |  |  |  |  |
| Item 3 | .52 | .60 |  |  |  |  |
| Item 4 | -.03 | .01 | .00 |  |  |  |
| Item 5 | -.03 | -.01 | -.02 | .55 |  |  |
| Item 6 | -.04 | .02 | .01 | .52 | .59 |  |

*Note*. Items 1-3 assess *facts* in science. Items 4-6 assess *discourse* in science.

Table S4. *Interitem Correlations for Language*

|  | Item 1 | Item 2 | Item 3 | Item 4 | Item 5 | Item 6 |
| --- | --- | --- | --- | --- | --- | --- |
| Item 1 |  |  |  |  |  |  |
| Item 2 | .42 |  |  |  |  |  |
| Item 3 | .41 | .46 |  |  |  |  |
| Item 4 | .07 | .17 | .20 |  |  |  |
| Item 5 | .06 | .13 | .15 | .55 |  |  |
| Item 6 | .08 | .18 | .19 | .51 | .55 |  |

*Note*. Items 1-3 assess *facts* in language. Items 4-6 assess *discourse* in language.

Material S1. *Self-report items used to measure students’ beliefs about disciplinary school cultures*

*Facts*

Item 1: In *subject* [science/ language] lessons, I am expected to know the content by heart.

Item 2: In *subject* [science/language] lessons, it is important to use technical terms for descriptions or justifications.

Item 3: In *subject* [science/language] lessons, I am expected to know specific terms.

*Discourse*

Item 4: In *subject* [science/language] lessons, I am expected to express my opinion about controversial issues.

Item 5: In *subject* [science/language] lessons, I am expected to discuss debatable topics.

Item 6: In *subject* [science/language] lessons, it is important to support my personal opinion about controversial issues.
